# Propofol attenuates the increase of sonographic optic nerve sheath diameter during robot-assisted laparoscopic prostatectomy: a randomized clinical trial

**DOI:** 10.1186/s12871-018-0523-7

**Published:** 2018-06-20

**Authors:** Jihion Yu, Jun Hyuk Hong, Jun-Young Park, Jai-Hyun Hwang, Seong-Sik Cho, Young-Kug Kim

**Affiliations:** 1 Department of Anesthesiology and Pain Medicine, Asan Medical Center, University of Ulsan College of Medicine, 88, Olympic-ro 43-gil, Songpa-gu, Seoul, 05505 Republic of Korea; 2 Department of Urology, Asan Medical Center, University of Ulsan College of Medicine, Seoul, 05505 Republic of Korea; 3 Department of Occupational and Environmental Medicine, Hallym University Sacred Heart Hospital, 22, Kwanpying-ro 170-gil, Dongan-gu, Anyang, 14068 Republic of Korea

**Keywords:** Propofol, Sevoflurane, Sonographic optic nerve sheath diameter, Robot-assisted laparoscopic prostatectomy

## Abstract

**Background:**

Robot-assisted laparoscopic prostatectomy (RALP) requires pneumoperitoneum and the Trendelenburg position to optimize surgical exposure, which can increase intracranial pressure (ICP). Anesthetic agents also influence ICP. We compared the effects of propofol and sevoflurane on sonographic optic nerve sheath diameter (ONSD) as a surrogate for ICP in prostate cancer patients who underwent RALP.

**Methods:**

Thirty-six patients were randomly allocated to groups receiving propofol (propofol group, *n* = 18) or sevoflurane (sevoflurane group, n = 18) anesthesia. The ONSD was measured 10 min after induction of anesthesia in the supine position (T1); 5 min (T2), 30 min (T3), and 60 min (T4) after establishing pneumoperitoneum and the Trendelenburg position; and at the end of surgery after desufflation in the supine position (T5). Respiratory and hemodynamic variables were also evaluated.

**Results:**

The ONSD was significantly different between the propofol group and the sevoflurane group at T4 (5.27 ± 0.35 mm vs. 5.57 ± 0.28 mm, *P* = 0.007), but not at other time points. The ONSDs at T2, T3, T4, and T5 were significantly greater than at T1 in both groups (all *P* < 0.001). Arterial carbon dioxide partial pressure, arterial oxygen partial pressure, peak airway pressure, plateau airway pressure, systolic blood pressure, pulse pressure variation, body temperature and regional cerebral oxygen saturation, except heart rate, were not significantly different between the two groups.

**Conclusions:**

The ONSD was significantly lower during propofol anesthesia than during sevoflurane anesthesia 60 min after pneumoperitoneum and the Trendelenburg position, suggesting that propofol anesthesia may help minimize ICP changes in robotic prostatectomy patients.

**Trial registration:**

Clinicaltrials.gov identifier: NCT03271502. Registered August 31, 2017.

## Background

Since robot-assisted laparoscopic prostatectomy (RALP) was first reported by Binder and Kramer in 2001, it has become the dominant surgical approach for prostate cancer treatment [1]. RALP has many benefits related to oncological outcomes and perioperative complications when compared to open surgery. These benefits include more precise manipulation of vessels and nerves; decreased blood loss; reduced surgical time, length of hospital stay, and postoperative pain; and improved quality of life [2, 3]. However, RALP requires carbon dioxide pneumoperitoneum and a steep Trendelenburg position to optimize surgical exposure. These conditions increase intracranial pressure (ICP) [4, 5]. Increased ICP and the resulting decreased cerebral perfusion pressure are associated with postoperative neurological complications such as cerebral ischemia and cerebrovascular disorders [6]. Therefore, it is necessary to minimize intraoperative ICP changes in robotic prostatectomy patients in whom ICP may increase beyond the normal range.

Anesthetic agents can influence ICP during surgery. In propofol anesthesia, a dose-related decrease in cerebral blood flow, cerebral metabolic rate, and ICP have been reported [7, 8]. Sevoflurane is a cerebral vasodilator with the potential to increase cerebral blood flow, cerebral blood volume, and ICP in a dose-dependent manner [9, 10]. However, the effects of anesthetics on ICP during carbon dioxide pneumoperitoneum and a steep Trendelenburg position during RALP have not been studied.

The purpose of this study was to compare the effects of propofol and sevoflurane on ICP in prostate cancer patients who underwent RALP. We evaluated the sonographic optic nerve sheath diameter (ONSD) as a surrogate for ICP [11].

## Methods

This randomized clinical trial included 36 patients who underwent RALP at Asan Medical Center during September 2017. Ethical approval for this study was provided by the Institutional Review Board at Asan Medical Center, Seoul, Republic of Korea (approval no. 2017–1011) on August 24, 2017. This study was registered with ClinicalTrials.gov (NCT03271502). Written informed consent was obtained from all patients.

### Patients

Patients who were scheduled for an elective RALP using the da Vinci™ robot system (Intuitive Surgical, Inc., Sunnyvale, CA, USA) were enrolled in this study. Patients with a history of cerebrovascular disease, those who refused to participate, and those younger than 20 or older than 79 were excluded.

### Randomization and interventions

Patients were randomly assigned to two groups using web-based randomization software (Random Allocation Software version 1.0, Isfahan University of Medical Sciences, Isfahan, Iran) [12]. We used block randomization with random block sizes of 6 and an allocation ratio of 1:1. One investigator kept sealed envelopes labeled with sequential study numbers, which were opened just before induction of anesthesia. The investigator performed total intravenous anesthesia with propofol (propofol group) or inhalation anesthesia with sevoflurane (sevoflurane group) according to a randomized table. The ventilator screen and the syringes of medications are concealed. We also prepared concealed syringes of normal saline in the sevoflurane group, indistinguishable from the outside. The investigators who measured the parameters did not know the method of anesthesia. The investigators who analyzed the data did not know the group. The investigators were blinded to the allocation of treatment until data analysis was complete.

### Study protocol

After routine hemodynamic monitoring including electrocardiogram, non-invasive blood pressure, pulse oximetry, bispectral index (Aspect Medical Systems, Inc., Newton, MA, USA) and regional cerebral oxygen saturation using near-infrared spectroscopy (IVOS 5100™, Somanetics Corp., Troy, MI, USA) were performed, preoxygenation was performed by administering 8 L/min of oxygen via facial mask before induction of anesthesia. Patients were randomly allocated into one of two groups according to a random table: the propofol group or the sevoflurane group. In the propofol group, propofol and remifentanil were infused continuously using a target-controlled infusion system to induce and maintain anesthesia. Propofol was adjusted to effect site target concentration of 1.5–3 μg/mL according to Marsh et al. [13]. Remifentanil was adjusted to effect site target concentration of 2–10 ng/mL according to Minto et al. [14]. In the sevoflurane group, anesthesia was induced with 1.5 mg/kg propofol and maintained with 1–2 vol% sevoflurane and continuous infusion of remifentanil into the effect site to a target concentration of 2–10 ng/mL, according to Minto et al. [14].

In the both groups, 0.6 mg/kg of rocuronium was administered for muscle relaxation during anesthetic induction. In addition, 0.1–0.2 mg/kg of rocuronium was intermittently administered during surgery. During the operation, propofol, sevoflurane, and remifentanil were adjusted to maintain a bispectral index score of 40–60, and arterial blood pressure and heart rate within 20% of the baseline. An arterial line was placed in the radial artery to continuously monitor the arterial blood pressure. Mechanical ventilation was set to volume control mode and a tidal volume of 8 mL/kg of ideal body weight. A respiratory rate of 10–20 breaths/min was adjusted to maintain an end-tidal carbon dioxide concentration of 30–35 mmHg. Positive end expiratory pressure was not applied. Oxygen at 50% was supplied using medical air. Plasma solution A (CJ Pharmaceutical, Seoul, Korea) as a crystalloid fluid was administered at a rate of 2–4 mL/kg/hr.

### Measurement

The ONSD was measured by investigators trained in ocular sonography. Measurements were performed in the transverse and sagittal planes of both eyes; the final ONSD value was the average of the 4 measured values. A 7.5-MHz linear probe was used for ONSD measurement, which was measured at 5 time points. The ONSD was measured at 10 min after anesthetic induction in the supine position (T1), 5 min after establishing carbon dioxide pneumoperitoneum and a steep Trendelenburg position (45-degree incline) (T2), 30 min after establishing carbon dioxide pneumoperitoneum and a steep Trendelenburg position (T3), 60 min after establishing carbon dioxide pneumoperitoneum and a steep Trendelenburg position (T4), and at the end of surgery after desufflation of pneumoperitoneum in the supine position (T5).

At each time point, we also measured the following variables: arterial carbon dioxide partial pressure (PaCO_2_), arterial oxygen partial pressure (PaO_2_), peak airway pressure, plateau airway pressure, systolic blood pressure, heart rate, pulse pressure variation, body temperature and regional cerebral oxygen saturation.

The duration of hospital stay and postoperative neurologic complications such as stroke, transient ischemic attack, and reversible neurologic deficit were evaluated.

### Statistical analysis

In the previous study, the mean ± standard deviation of ONSD was 4.9 ± 0.4 mm during pneumoperitoneum and the Trendelenburg position under sevoflurane anesthesia [15]. Assuming that the ONSD of propofol anesthesia was reduced by 10% compared with the ONSD of sevoflurane anesthesia, the mean difference of the ONSD between propofol anesthesia and sevoflurane anesthesia was 0.49 mm. The sample size was calculated to be at least 16 subjects for each group, using a power of 90% and at a significance level of *P* < 0.05. Considering a dropout rate of 10%, the total number of patients was 36, or 18 patients per group. All data are expressed as the mean ± standard deviation or as number (%). Comparisons between the groups were performed using a Student’s t-test, Mann-Whitney U test, chi-square test, or Fisher’s exact test, as appropriate. A two-way repeated measures analysis of variance with Bonferroni post-testing was used to compare the ONSD, respiratory variables, and hemodynamic variables within and between the two groups. *P* values < 0.05 were considered significant. All statistical analyses were performed using IBM SPSS Statistics for Windows, Version 22.0 (IBM Corp., Armonk, NY, USA).

## Results

**Fig. 1:**
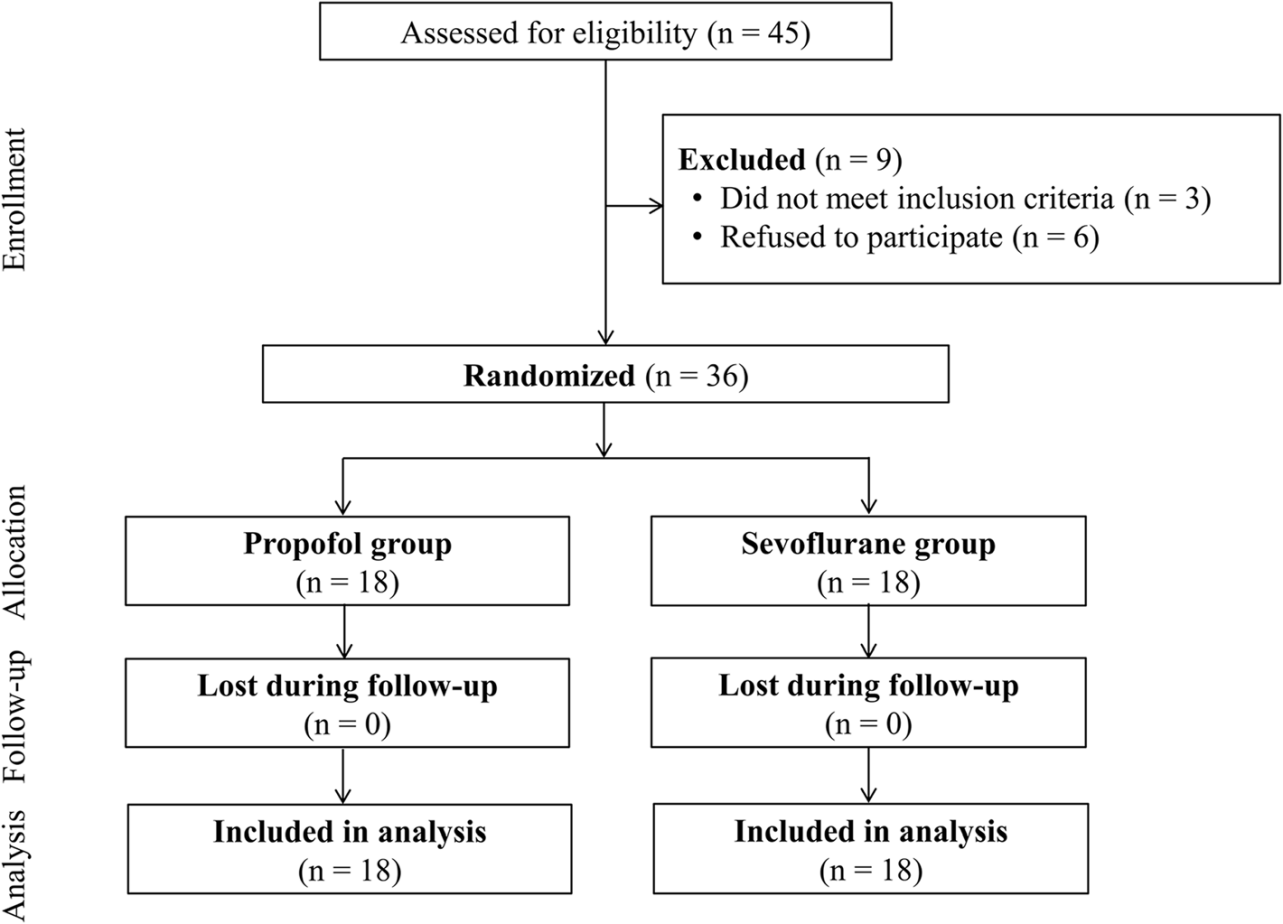
Study flow diagram

During the study period, 45 patients were screened. Three patients were excluded due to a history of cerebrovascular disease and six patients refused to participate. A total of 36 patients were included and were randomly allocated to either the propofol group or the sevoflurane group (Fig. 1). Demographic and intraoperative data were not significantly different between the two groups (Table 1).

**Table 1:** Demographic and intraoperative data

Variables	All patients (*n* = 36)	Propofol group (*n* = 18)	Sevoflurane group (n = 18)	P value^*^
Age (yr)	64.8 ± 7.6	66.1 ± 7.2	63.6 ± 7.9	0.339
Weight (kg)	71.1 ± 8.7	72.3 ± 6.5	69.8 ± 10.6	0.399
Height (cm)	165.8 ± 6.0	166.0 ± 4.0	165.6 ± 7.6	0.868
Body mass index (kg/m^2^)	25.8 ± 2.3	26.1 ± 1.9	25.4 ± 2.7	0.410
Hypertension	16 (44.4%)	8 (44.4%)	8 (44.4%)	1.000
Diabetes mellitus	4 (11.1%)	3 (16.7%)	1 (5.6%)	0.289
Preoperative laboratory values				
Hemoglobin (g/dL)	14.2 ± 0.9	13.9 ± 0.9	14.5 ± 0.9	0.060
Albumin (g/dL)	3.88 ± 0.24	3.93 ± 0.18	3.83 ± 0.28	0.211
Creatinine (mg/dL)	0.90 ± 0.22	0.92 ± 0.28	0.87 ± 0.13	0.478
Operation time (min)	147.1 ± 23.4	149.9 ± 20.2	144.2 ± 26.6	0.472
Anesthesia time (min)	187.8 ± 22.9	190.6 ± 18.9	184.9 ± 26.6	0.461
Crystalloid amount (mL)	1273.6 ± 318.8	1227.8 ± 351.1	1319.4 ± 285.5	0.396

Data are presented as mean ± standard deviation or as number (%)

* indicates comparison between the propofol group and the sevoflurane group

**Fig. 2:**
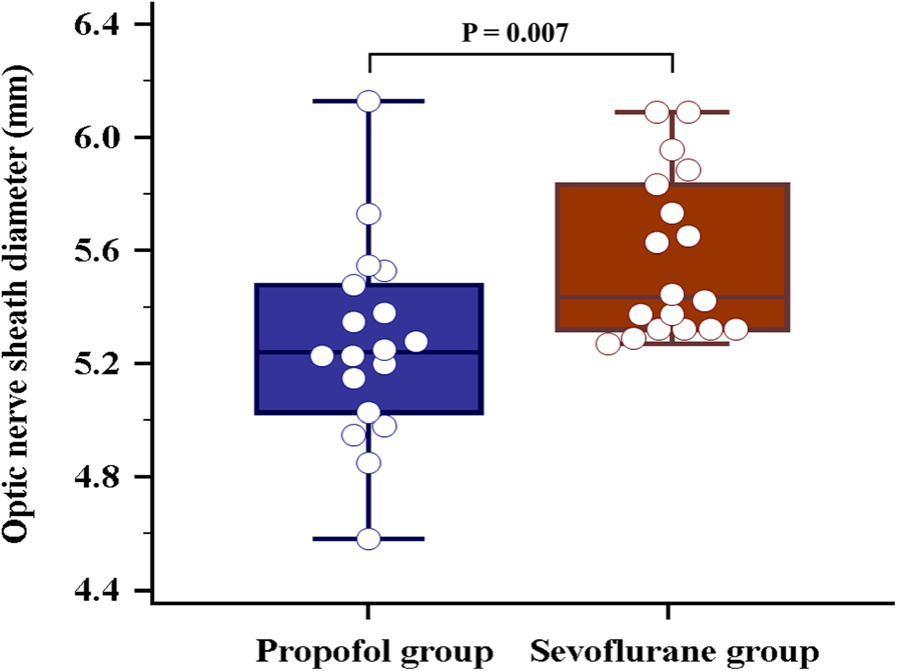
Comparison of the optic nerve sheath diameters between the propofol group and the sevoflurane group at 60 min after carbon dioxide pneumoperitoneum and a steep Trendelenburg position

The ONSDs of the two groups are shown in Table 2. The ONSDs were not significantly different between the two groups 10 min after anesthetic induction in the supine position (T1) or 5 min after establishing carbon dioxide pneumoperitoneum and steep Trendelenburg position (T2). However, 30 min after carbon dioxide pneumoperitoneum and steep Trendelenburg position (T3), the ONSD tended to differ between the groups (5.22 ± 0.34 mm vs. 5.42 ± 0.36 mm, *P* = 0.096). Sixty minutes after carbon dioxide pneumoperitoneum and a steep Trendelenburg position (T4), the ONSD was significantly different between the two groups (5.27 ± 0.35 mm vs. 5.57 ± 0.28 mm, *P* = 0.007) (Fig. 2). There were significant increases in the ONSDs in both groups at T2, T3, and T4 compared to T1 (propofol group: all *P* < 0.001; sevoflurane group: all P < 0.001). During pneumoperitoneum and the Trendelenburg position at T2, T3, and T4, sonographic ONSDs tended to increase continuously (Table 2).

**Table 2:** Sonographic optic nerve sheath diameters in the propofol group and the sevoflurane group during robot-assisted laparoscopic prostatectomy

Time point	Propofol group (mm)	Sevoflurane group (mm)	P value
T1	4.75 ± 0.37	4.74 ± 0.42	0.942
T2	5.09 ± 0.36	5.22 ± 0.41	0.319
T3	5.22 ± 0.34	5.42 ± 0.36	0.096
T4	5.27 ± 0.35	5.57 ± 0.28	0.007
T5	5.18 ± 0.37	5.29 ± 0.41	0.403

Data are presented as mean ± standard deviation. *T1 =* 10 min after anesthetic induction in the supine position, *T2 =* 5 min after establishing carbon dioxide pneumoperitoneum and a steep Trendelenburg position, *T3 =* 30 min after establishing carbon dioxide pneumoperitoneum and a steep Trendelenburg position, *T4 =* 60 min after establishing carbon dioxide pneumoperitoneum and a steep Trendelenburg position, *T5 =* at the end of surgery after desufflation of pneumoperitoneum in the supine position

**Fig. 3:**
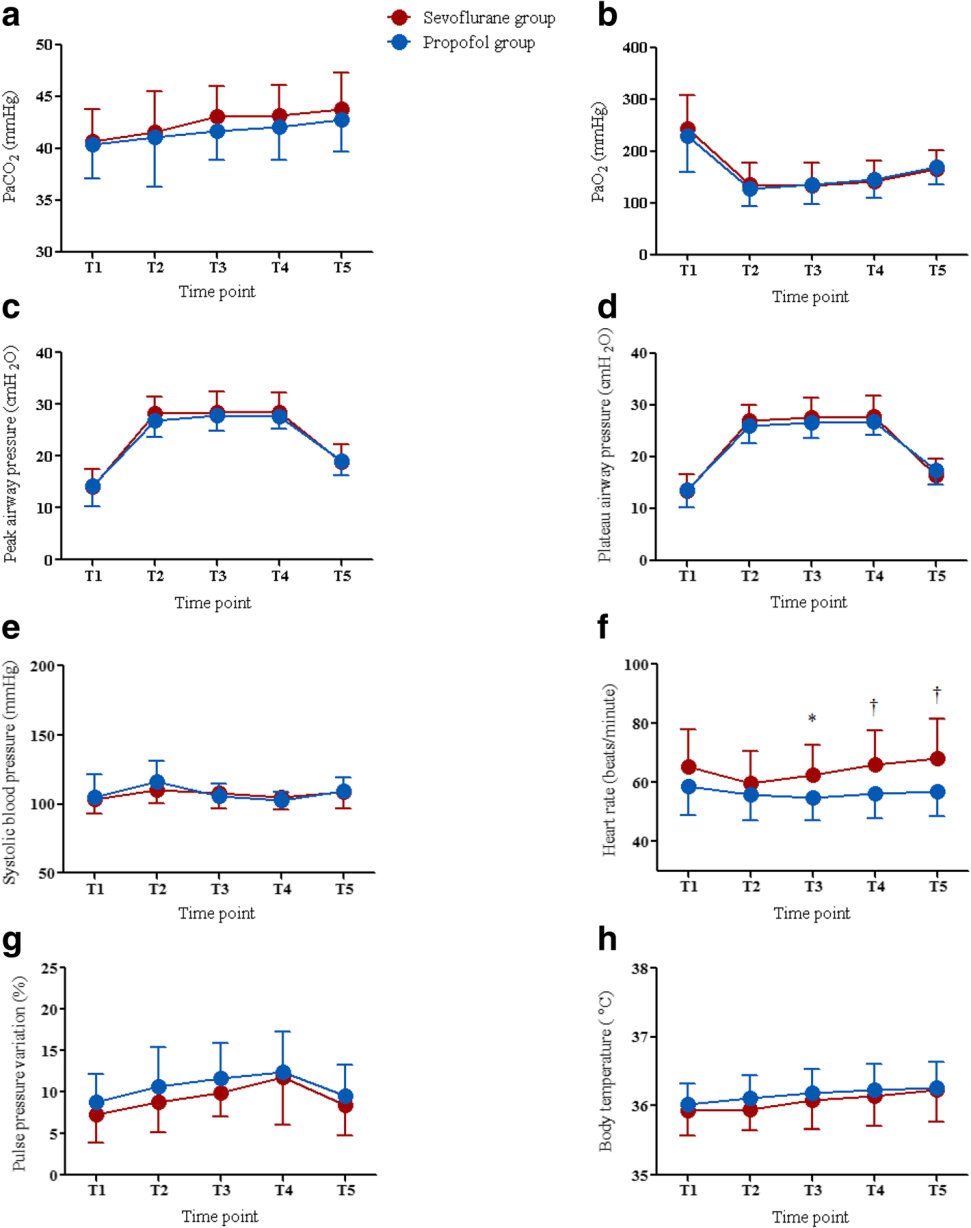
Comparisons of PaO_2_ (**a**) PaCO_2_ (**b**) peak airway pressure (**c**) plateau airway pressure (**d** systolic blood pressure (**e**) heart rate (**f**) pulse pressure variation (**g**) and body temperature (**h**) between the propofol group (blue circle) and the sevoflurane group (red circle) during robot-assisted laparoscopic prostatectomy. Note that all parameters except heart rate do not significantly differ between the two groups. PaCO_2_ = arterial carbon dioxide partial pressure, PaO_2_ = arterial oxygen partial pressure, T1 = 10 min after anesthetic induction in the supine position, T2 = 5 min after establishing carbon dioxide pneumoperitoneum and a steep Trendelenburg position, T3 = 30 min after establishing carbon dioxide pneumoperitoneum and a steep Trendelenburg position, T4 = 60 min after establishing carbon dioxide pneumoperitoneum and a steep Trendelenburg position, and T5 = at the end of surgery after desufflation of pneumoperitoneum in the supine position. * indicates *P* < 0.05 between the two groups; † indicates *P* < 0.01 between the two groups

There were no significant differences in PaCO_2_, PaO_2_, peak airway pressure, plateau airway pressure, systolic blood pressure, pulse pressure variation, and body temperature between the groups (Fig. 3). However, heart rate was higher at T3, T4, and T5 in the sevoflurane group than in the propofol group (T3, *P* = 0.018; T4, *P* = 0.006; T5, *P* = 0.005). In addition, there were no significant differences in regional cerebral oxygen saturation between the propofol group and the sevoflurane group at all predetermined time points under general anesthesia (66.3% vs. 67.7%, *P* = 0.539 at T1; 65.1% vs. 66.1%, *P* = 0.614 at T2; 64.4% vs. 65.3%, *P* = 0.698 at T3; 64.3% vs. 65.9%, *P* = 0.470 at T4; 65.1% vs. 68.4%, *P* = 0.152 at T5).

There was no significant difference in the duration of hospital stay between the propofol group and the sevoflurane group (7.7 days vs. 7.6 days, *P* = 0.635). In addition, postoperative neurologic complications did not occur in both groups.

## Discussion

The sonographic ONSD of propofol anesthesia was significantly lower than that of sevoflurane anesthesia 60 min after establishing carbon dioxide pneumoperitoneum and a steep Trendelenburg position in prostate cancer patients who underwent RALP. In addition, the sonographic ONSDs increase continuously after establishing carbon dioxide pneumoperitoneum and a steep Trendelenburg position in both groups.

Anesthetics suppress the activity of electroencephalogram and reduce the cerebral metabolic rate. However, changes in cerebral metabolic rate and cerebral blood flow are inconstant and depends on how the anesthetic affects the cerebral vascular smooth muscle [16]. Sevoflurane has a dose-dependent effect on the vascular smooth muscle relaxation and intrinsic cerebral vasodilatory activity through the direct inhibition of several pathways. Therefore, cerebral blood flow increases significantly during sevoflurane anesthesia, and ICP can increase as a result [10, 17, 18]. However, propofol slows the activity of electroencephalogram, decreases the rate of consumption of adenosine, and reduces the cerebral metabolic rate [8, 19, 20]. Decreased cerebral metabolic rate reduces cerebral blood perfusion, which is known to reduce cerebral blood flow and ICP [21]. In addition, previous human and animal studies determined that propofol led to a progressive decrease in ICP [22–24]. In particular, ICP tended to decrease over time during the continuous administration of propofol. Farling et al. continuously administered propofol to head injury patients in intensive care and measured the ICP at multiple time points. They reported that ICP significantly decreased after 2 h of continuous administration [23]. Therefore, it could be expected that the difference in ICP between the two groups would gradually increase over time. In the present study, the difference in the ONSD between the two groups was not statistically significant at T2, but tended to differ between groups at T3. The ONSD at T4 was significantly different between the two groups. We consider that the continuous administration of propofol had a beneficial effect on ICP during RALP.

Many studies have determined that carbon dioxide pneumoperitoneum and a steep Trendelenburg position could increase ONSD as a surrogate for ICP [15, 25–27]. The pneumoperitoneum and Trendelenburg position may disturb cerebral venous drainage due to increased intrathoracic pressure, and may lead to a subsequent increase in ICP [28–30]. Moreover, carbon dioxide can increase intracranial vascular perfusion and elevate ICP [31–33]. In the present study, ONSD increased steadily from the induction of carbon dioxide pneumoperitoneum and steep Trendelenburg position in both groups. A previous study reported that ONSD increased immediately after establishing pneumoperitoneum and a steep Trendelenburg position, but then remained unchanged [25]. The ongoing increase in ONSD in our study is thought to be due at least in part to an age-related difference. Based on a previous study regarding ONSD change in patients during RALP [34], the ONSD tended to increase continuously over time in patients greater than 63 years of age after establishing pneumoperitoneum and a steep Trendelenburg position. However, in patients less than 63 years of age, ONSD decreased over the course of pneumoperitoneum and a steep Trendelenburg position [34]. The authors explained that younger patients had better autoregulation of ONSD or ICP during pneumoperitoneum and the Trendelenburg position. In the present study, the average age of the propofol group was 66.1 ± 7.2 years, and that of the sevoflurane group was 63.6 ± 7.9 years. Therefore, it seems likely that the ability to compensate for ICP changes might be reduced, and so ONSD increased continuously over time during surgery.

ONSD measurement using ultrasonography is a simple, non-invasive technique for ICP assessment. Previous studies have reported that sonographic measurement of ONSD strongly correlates with measurement performed by inserting an invasive catheter into one of the lateral ventricles or the brain parenchyma in various clinical situations, including RALP [11, 15, 35, 36]. Intracranial hypertension is defined as ICP greater than 20 mmHg [37], and the cut off value of the ONSD for intracranial hypertension is known to be 4.8–5.2 mm [38–41]. In the present study, ONSD was measured as 5.27 ± 0.35 mm in the propofol group and 5.57 ± 0.28 mm in the sevoflurane group at 60 min after establishing pneumoperitoneum and the Trendelenburg position. Because this is a situation in which intracranial hypertension might be suspected, it is important to minimize the increase in ONSD during RALP.

The present study has the following limitations. We could not measure ICP because the difficulty and invasiveness of the procedure made it impossible to be carried out in non-neurosurgical patients. However, sonographic measurement of ONSD is a non-invasive technique and has been shown to accurately reflect increases in ICP [42, 43]. Also, we did ONSD was not measured immediately after establishing carbon dioxide pneumoperitoneum and a steep Trendelenburg position. Instead, ONSD was measured at 5 min after establishing pneumoperitoneum and the Trendelenburg position, a time point that can adequately reflect the immediate change of ONSD during RALP.

## Conclusion

We found that ONSD was significantly lower during propofol anesthesia than during sevoflurane anesthesia 60 min after carbon dioxide pneumoperitoneum and a steep Trendelenburg position in robotic prostatectomy patients. Our results suggest that propofol anesthesia might be used effectively to minimize intraoperative ICP changes in prostate cancer patients who are undergoing RALP using pneumoperitoneum and the Trendelenburg position.

## Abbreviations

ICP: Intracranial pressure
ONSD: Optic sheath nerve diameter
PaCO_2_: Arterial carbon dioxide partial pressure
PaO_2_: Arterial oxygen partial pressure
RALP: Robot-assisted laparoscopic prostatectomy
T1: 10 min after anesthetic induction in the supine position,
T2: 5 min after establishing carbon dioxide pneumoperitoneum and a steep Trendelenburg position (45-degree incline)
T3: 30 min after establishing carbon dioxide pneumoperitoneum and a steep Trendelenburg position
T4: 60 min after establishing carbon dioxide pneumoperitoneum and a steep Trendelenburg position
T5: At the end of surgery after desufflation of pneumoperitoneum in the supine position
